# Differential Responses of Wheat (*Triticum aestivum* L.) and Cotton (*Gossypium hirsutum* L.) to Nitrogen Deficiency in the Root Morpho-Physiological Characteristics and Potential MicroRNA-Mediated Mechanisms

**DOI:** 10.3389/fpls.2022.928229

**Published:** 2022-06-30

**Authors:** Huiyun Xue, Jia Liu, Sando Oo, Caitlin Patterson, Wanying Liu, Qian Li, Guo Wang, Lijie Li, Zhiyong Zhang, Xiaoping Pan, Baohong Zhang

**Affiliations:** ^1^Henan Collaborative Innovation Center of Modern Biological Breeding, Henan Institute of Science and Technology, Xinxiang, China; ^2^Department of Biology, East Carolina University, Greenville, NC, United States; ^3^Department of Biology, Elizabeth City State University, Elizabeth City, NC, United States; ^4^College of Life Sciences, Anhui Normal University, Wuhu, China

**Keywords:** wheat, cotton, root, microRNA, nitrogen deficiency, physiology

## Abstract

Understanding the mechanism of crop response to nitrogen (N) deficiency is very important for developing sustainable agriculture. In addition, it is unclear if the microRNA-mediated mechanism related to root growth complies with a common mechanism in monocots and dicots under N deficiency. Therefore, the root morpho-physiological characteristics and microRNA-mediated mechanisms were studied under N deficiency in wheat (*Triticum aestivum* L.) and cotton (*Gossypium hirsutum* L.). For both crops, shoot dry weight, plant dry weight and total leaf area as well as some physiological traits, i.e., the oxygen consuming rate in leaf and root, the performance index based on light energy absorption were significantly decreased after 8 days of N deficiency. Although N deficiency did not significantly impact the root biomass, an obvious change on the root morphological traits was observed in both wheat and cotton. After 8 days of treatment with N deficiency, the total root length, root surface area, root volume of both crops showed an opposite trend with significantly decreasing in wheat but significantly increasing in cotton, while the lateral root density was significantly increased in wheat but significantly decreased in cotton. At the same time, the seminal root length in wheat and the primary root length in cotton were increased after 8 days of N deficiency treatment. Additionally, the two crops had different root regulatory mechanisms of microRNAs (miRNAs) to N deficiency. In wheat, the expressions of miR167, miR319, miR390, miR827, miR847, and miR165/166 were induced by N treatment; these miRNAs inhibited the total root growth but promoted the seminal roots growth and lateral root formation to tolerate N deficiency. In cotton, the expressions of miR156, miR167, miR171, miR172, miR390, miR396 were induced and the expressions of miR162 and miR393 were inhibited; which contributed to increasing in the total root length and primary root growth and to decreasing in the lateral root formation to adapt the N deficiency. In conclusion, N deficiency significantly affected the morpho-physiological characteristics of roots that were regulated by miRNAs, but the miRNA-mediated mechanisms were different in wheat and cotton.

## Introduction

Nitrogen (N) is one of the most abundant nutrients for plant growth. The application of N fertilizer has always been a key way to increase crop yields (Zhang, 2007; Gao et al., 2015; Shang et al., 2015). Unfortunately, the usage efficiency of N fertilizer is relatively low, because the N fertilizer is easy to volatilize, leach, and denitrify in the soil; over usage of N fertilizer also results in many environmental problems, such as the eutrophication of water and deterioration of air quality (Carpenter et al., 1998; Stulen, 1998; Tilman, 1999; Richter and Roelcke, 2000; Xing and Zhu, 2000). Therefore, understanding the mechanism of N-deficiency tolerance in crops is very important for optimizing N management, breeding new cultivars with high N usage efficiency and further developing sustainable agriculture.

Root system is the main organ for absorbing water and nutrients (Lynch, 2013; Shang et al., 2015). Root architectural, morphological phenes, and phene aggregates affect the utilization of soil resource (York and Lynch, 2015). For example, maize (*Zea mays*) plants with few but long lateral roots usually have greater N acquisition than that with many but short lateral roots, because of deeper rooting and greater axial root elongation (Zhan and Lynch, 2015). In addition, root architectural and morphological phenes have a high plasticity with the availability of soil resource (Asseng et al., 1998; Sun et al., 2017). Linkohr et al. (2002) compared the change of root system architecture of *Arabidopsis* in homogeneous, heterogeneous provision of nitrate and phosphate, founded that the primary root length was decreased with increasing availability of nitrate, while increased with more supply of phosphate. Therefore, root systems play a key role in environmental fitness, crop performance and yield (Shang et al., 2015). Plants have evolved several strategies to adapt N deficiency through the morphological, physiological, biochemical, or molecular processes (Chuck et al., 2007; Gao et al., 2018), which includes increasing the length and number of fine roots or decreasing the stele diameter and tracheid diameter of coarse roots (Zhang et al., 2011), altering the root architecture to have the largest surface area for absorbing soil resource (Engineer and Kranz, 2007), optimizing the N allocation (Gao et al., 2015), increasing the enzyme activities for N assimilation (Fuentes et al., 2001; Feng et al., 2016), and improving ability of N assimilation by regulating the genes involved in C metabolism (Yanagisawa et al., 2004; Feng et al., 2016). However, there is a significant difference between monocot plants and dicot plants in root morphological, structural characters, and biochemical process. The roots of almost all monocots have a fibrous root system which is characterized by many adventitious roots, while the roots of most dicots have a tap root system including the primary root and lateral roots (Osmont et al., 2007; Bellini et al., 2014). The conserved function of GRAS gene family in regulating root radial patterning of all plant species (Cui et al., 2007; Hochholdinger and Zimmermann, 2008), the diverse function of lateral organ boundaries (LOB) domain proteins for root formation in maize and *Arabidopsis* (Hochholdinger and Zimmermann, 2008), and the histological differences of root in *Arabidopsis* and cereals (Schiefelbein et al., 1997; Jiang et al., 2003).

MicroRNAs (miRNAs) are an abundant class of endogenous noncoding RNAs with 21–24 nucleotides in length, which regulate the target genes by inhibiting mRNA translation or splicing the mRNAs that have complementary base-pairing with the miRNA (Zhang et al., 2006a; Wang et al., 2017); therefore miRNAs regulate the development and stress responses in animals and plants (Bartel, 2009; Voinnet, 2009; Zhang, 2015). Studies also show that miRNAs regulated root growth and development (Meng et al., 2010; Khan et al., 2011; Li and Zhang, 2016), including embryonic root development, primary root growth, root radial patterning, tissue differentiation, lateral root, and adventitious root development. By targeting PXMT1, overexpression of miR163 inhibited primary root elongation (Li T. et al., 2021). miR393 with a si-TAAR suppressed primary root elongation which was involved in the abscisic acid (ABA) signaling pathway (Chen et al., 2012; Yan et al., 2022); miR393 suppressed root growth by targeting AFB3 in *Arabidopsis* (Vidal et al., 2010). Many of miRNAs are proved to be highly conserved throughout plant evolution by regulate conserved target genes (Zhang et al., 2006b). For example, the miR164-NAC (NAMATAF-CUC) module, miR156-SPLs (SQUAMOSA PROMOTER BINDING-LIKE) module and miR396-GRFs (GROWTH RESPONSE FACTORS) are conserved across plant species in regulating plant growth (Willmann and Poethig, 2007). Several miRNAs have been reported in association with N stresses under different tissue (Chiou, 2010; Zhao et al., 2011; Zeng et al., 2014). Through genome-wide identification, miRNAs including 119 conserved and 1,002 putative novel were identified in potato under N deficiency (Tiwar et al., 2020).

Although many experiments have been done on root development under N deficiency, more systematic investigations are still essential, particularly on the comparison between monocots and dicots under N deficiency. In addition, it is unclear if the miRNA-mediated mechanism related to root growth complies with a common mechanism in monocots and dicots under N deficiency. Wheat (*Triticum aestivum* L.) as one of the most important monocot crops, supplied about 35% of staple food for the world’s population. While cotton (*Gossypium hirsutum* L.), one dicot, is an important cash crop in the world, contributed greatly to the industrial and economic development in many countries (Hakoomat et al., 2014). In this study, we systematically analyzed the change of root morphological characteristics and physiological feature during sustained N deficiency in wheat and cotton. We also explored the regulate mechanisms of miRNAs in root growth in wheat and cotton under N deficiency. This research provided a new strategy to improve the N use efficiency and cultivate N efficient varieties of wheat and cotton by regulating the expression of miRNAs and their targeted mRNAs under N deficiency.

## Materials and Methods

### Seed Germination and Sampling

Wheat cultivar AK-58 and cotton cultivar Baimian No. 1 were used in this research; both cultivars were widely cultivated in China. Healthy mature wheat and cotton seeds with same size were selected and surface sterilized in 10% H_2_O_2_ for 5 min and 20 min, respectively, followed by washing for five times with sterilized water. Then, the sterilized seeds were germinated and cultivated as described in our previous studies (Fontana et al., 2020; Thornburg et al., 2020). In order to analyze the effect of N, two N concentrations were provided. The normal N (NN) solution served as the control, which consisted of: 2.5 mM Ca(NO_3_)_2_, 1 mM MgSO_4_, 0.5 mM KH_2_PO_4_, 2 mM NaCl, 2 mM KCl, 0.1 mM EDTA-FeNa, 0.2 μM CuSO_4_, 1 μM ZnSO_4_, 0.02 mM H_3_BO_3_, 5 nM (NH4)_6_Mo_7_O_24_, 1 μM MnSO_4_. While the low N (LN) solution consisted of 0.05 mM Ca(NO_3_)_2_ and 2.45mM CaCl_2_ with other element concentration at the same with the control. After 4 and 8 days of N stress treatments, the wheat and cotton seedlings of each treatment were sampled for measuring the morphological traits, physiological traits, and the expression of miRNAs and their targeted mRNA genes.

### Morphology Analysis and Biomass Measurement of Wheat and Cotton Plants

The shoot and roots were first separated from each plant; then, the fresh weights were weighed. The fresh tissues were dried at an oven with 80°C for 2 days and then the dried weight was measured.

The number and length of seminal roots of wheat, primary root of cotton were first measured manually. Then, each part was scanned by using the Epson scanner (Epsin America lnc., California, CA, United States), and saved with images. WinRHIZO was used to analyze the images, and the total length of root, surface area of leaf and root, root volume, and root average diameter per plant were measured.

Four and six biological replicates were performed for wheat and cotton, respectively, for each trait for each treatment.

### Measurement of Chlorophyll Fluorescence and Chlorophyll Content

The chlorophyll fluorescence of the first fully-opened leaves in wheat and the second fully opened leaves in cotton of each treatment were measured by using a portable fluorometer (Handy PEA, Hansatech, Norfolk, United Kingdom) with four to six biological replicates at the 4th and 8th days after N deficiency. Before measurements, the selected leaves were dark-adapted for 30 min. A fast fluorescence curves was induced by 1 s pulses of red light (650 nm, 3500 μmol⋅ m^–2^⋅ s^–1^). The performance index based on light energy absorption (PI_ABS_) that calculated from the fast fluorescence curves was used to reflect the photosynthetic capacity of leaves. After the measurement of chlorophyll fluorescence, the leaves were used to measure the chlorophyll content according to the method in our previous studies (Thornburg et al., 2020) at the 4th and 8th days after N deficiency treatments.

### Root Vitality, Root and Leaf Respiration Rate Measurement

Roots of four to six wheat and cotton plants under each treatment were selected to measure root vitality according to the Triphenyl tetrazolium chloride (TTC) method as described in our previous studies (Fontana et al., 2020; Thornburg et al., 2020). The respiration rate of four to six plant roots and leaves were also monitored as described in our previous studies (Fontana et al., 2020; Thornburg et al., 2020) by using a Clark Chlorolab2 system (Hansatech Instruments Ltd., Norfolk, United Kingdom).

### Extraction of RNAs and Gene Expression Analysis

Total RNAs were extracted by using the MirVana miRNA Isolation Kit (Ambion, Austin, TX, United States) from each sample collected at the 4th and 8th days after N deficiency treatment according to the manufacturer’s instructions The quality of RNAs was measured by Nanodrop ND-1000 (Nanodrop Technologies, Lnc, Oxfordshire, United States). Then, the extracted RNAs were stored at –80°C until further analysis. To reveal the miRNA-mediated mechanism under N deficiency, 20 miRNAs and their targets that associated with root development, nutrient stress in crops and reported in our previous studies were tested according to the method of previous reports (Fontana et al., 2020; Thornburg et al., 2020; Li L. et al., 2021). Three biological replicates were run for each treatment. Three technical replicates were run to avoid pipetting-related issue.

### Statistical Analysis

ANOVA test of the morphological index, physiological index, and gene expression between two treatments were performed by using SPSS version 22. Data presented in the figures and tables are means ± S.D. of three to six biological replicates.

## Results

### Effects of N Deficiency on the Plant Growth and the Root Morphological Traits in Wheat and Cotton Seedlings

**FIGURE 1:**
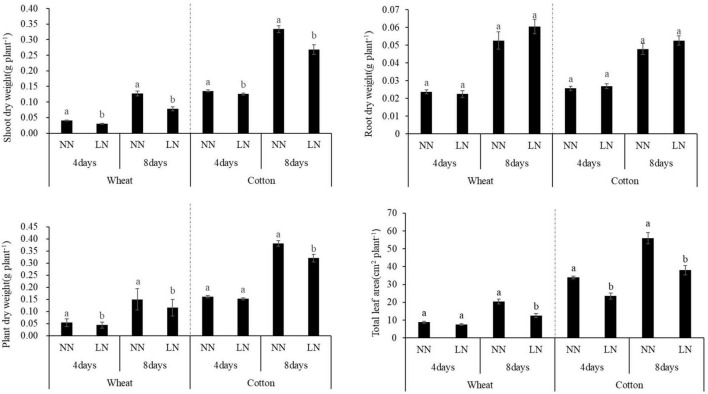
Effects of N deficiency on plant growth and development in wheat and cotton seedlings. Different lowercase letters above the bars mean significant differences between the control and the N deficiency treatment on the same time in wheat and cotton according to the LSD test (*p* = 0.05).

There was a significant impact on the growth of both crops under N deficiency treatments. Compared with the normal N treatment, there was a significant decrease in the shoot dry weight in both crops after 4 and 8 days of N deficiency treatments. In addition, a significant decrease in plant dry weight and total leaf area were observed for both crops after 4 and 8 days of N deficiency treatments with 23.06 and 38.82% of decrease in wheat, 15.94 and 32.12% of decrease in cotton compared with the control treatment. Although the root dry weight was increased to 14.83% in wheat and 10.04% in cotton seedling under N deficiency than that of the controls from 4 days to 8 days of treatments, no obvious difference was observed between the treatments (Figure 1).

**FIGURE 2:**
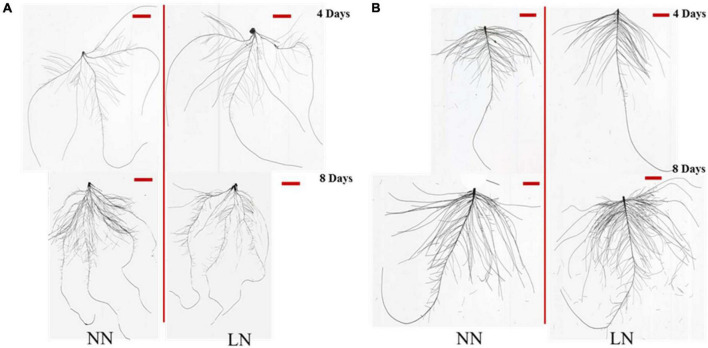
The root morphological traits of wheat **(A)** and cotton **(B)** seedlings between normal N (NN) and low N (LN) group after 4 and 8 days of treatment. The original size of the horizontal scale was 0.3 cm in height and 2.7 cm in width.

Further analysis of root morphological characteristics, obvious effects were observed under N deficiency (Table 1 and Figure 2). First, the total root length, root surface area, root volume area, root average diameter, lateral root density, seminal root length, or primary root length of both crops had no significant difference with the values of the normal treatment on the 4th days of treatments. After 8 days of treatments, the total root length, root surface area, and root volume of both crops had a significant difference between the N deficiency treatment and the control, with a decrease of 23.24, 24.45, and 26.56% in wheat, an increase of 41.57, 38.19, and 33.87% in cotton, respectively. Compared with the normal N treatment, after 8 days of treatment, there were an obvious increase on the seminal root length of wheat and primary root length of cotton under N deficiency increased by 21.26% and 32.11% in wheat and cotton, respectively. While only the primary root length of cotton reached a significant level under N deficiency compared with the normal N treatment on the 8th day. Effects of N deficiency were opposite on the lateral root density of the two crops, with the lateral root density of wheat significantly increased and the lateral root density of cotton significantly decreased after 8 days of treatment.

**TABLE 1:** Effects of N deficiency on the root morphological traits of wheat and cotton seedlings.

Crop	DAT (d)	Treatments	TRL (cm/plant)	RSA (cm^2^/plant)	RV (cm^3^/plant)	RAD (mm plant^–1^)	SRL/PRL (cm/plant)	LRD (No. cm^–1^)
Wheat	4	NN	401.96 ± 20.91a	34.61 ± 1.2a	0.24 ± 0.02a	0.27 ± 0.01a	105.78 ± 9.84a	6.51 ± 1.54a
		LN	408.75 ± 57.99a	35.57 ± 4.67a	0.25 ± 0.03a	0.28 ± 0.01a	101.70 ± 9.47a	6.05 ± 1.13a
	8	NN	1232.42 ± 173.05a	97.65 ± 11.05a	0.64 ± 0.06a	0.86 ± 0.08a	142.45 ± 9.52a	8.29 ± 1.02b
		LN	945.95 ± 111.17b	73.77 ± 7.96b	0.47 ± 0.07b	0.76 ± 0.03a	172.73 ± 34.64a	9.65 ± 0.23a
Cotton	4	NN	404.36 ± 49.41a	45.00 ± 5.48a	0.40 ± 0.05a	0.35 ± 0.01a	23.90 ± 1.00a	5.94 ± 1.59a
		LN	388.24 ± 45.44a	44.80 ± 468a	0.41 ± 0.06a	0.37 ± 0.03a	25.98 ± 4.39a	5.73 ± 1.19a
	8	NN	635.17 ± 104.60b	70.10 ± 9.97b	0.62 ± 0.08b	0.35 ± 0.01a	24.54 ± 3.44b	9.30 ± 1.88a
		LN	899.24 ± 82.29a	96.87 ± 10.63a	0.83 ± 0.12a	0.34 ± 0.01a	32.42 ± 0.87a	7.00 ± 0.17b

*Different lowercase letters in same column mean significant differences between the control treatment and N deficiency on the same time in wheat and cotton according to the LSD test (p = 0.05). TRL, Total root length; RSA, Root surface area; RV, Root volume; RAD, Root average diameter; SRL, Seminal root length; PRL, primary root length; LRD, Lateral root density.*

### Effects of N Deficiency on the Leaf and Root Physiological Traits of Wheat and Cotton Seedlings

After 8 days of treatment, the chlorophyll a, chlorophyll b, and total chlorophyll content were decreased for both crops under N deficiency compared with the normal N treatment, while only the chlorophyll a and total chlorophyll content in wheat had significant difference between the N deficiency and the control (Table 2). Consumption of oxygen in leaf is also an indicator for leaf vigor. Compared with the control treatment, there was a significant decrease (29.81%) of the O_2_ consuming rate in wheat leaves under N deficiency, while a significant increase (40.47%) was observed in cotton leaves on the 4th of N deficiency. After 8 days of treatments, the O_2_ consuming rates were decreased by 35.90% and 58.21%, in wheat and cotton, respectively, comparing with the control treatment (Table 2). One of the major symptoms of N deficiency was the decrease of photosynthesis. PI_ABS_ is a photosynthesis performance index that reflects the overall photosynthetic activity of the light reactions of photosystem II (PSII). Therefore, the Chlorophyll fluorescence parameter of PI_ABS_ was observed. Compared with the controls, there was a significant decrease of PI_ABS_ in both crops under N deficiency, the PI_ABS_ was decreased by 21.02 and 62.50%, in wheat; and it was decreased by 51.04 and 86.50% in cotton after 4 and 8 days of treatments, respectively. With the duration (4–8 days of treatment) of N deficiency, PI_ABS_ was decreased by 49.81% from 2.63 to 1.32 in wheat, decreased by 72.88% from 1.18 to 0.32 in cotton (Table 2).

**TABLE 2:** Effects of N deficiency on the leaf physiological traits of wheat and cotton seedlings.

Crop	DAT (d)	Treatments	Chlorophyll a (mg/g)	Chlorophyll b (mg/g)	Total chlorophyll content (mg/g)	O_2_ consuming rate of leaf (nM/min/g FW)	PI_ABS_
Wheat	4	NN	1.55 ± 0.56a	0.88 ± 0.73a	2.42 ± 1.26a	179.75 ± 13.54a	3.33 ± 0.13a
		LN	1.34 ± 0.79a	0.88 ± 0.96a	2.21 ± 1.73a	126.17 ± 20.61b	2.63 ± 0.12b
	8	NN	1.83 ± 0.32a	0.85 ± 0.42a	2.68 ± 0.71a	738.12 ± 26.27a	3.52 ± 0.15a
		LN	1.03 ± 0.34b	0.79 ± 0.46a	1.82 ± 0.80b	473.11 ± 83.02b	1.32 ± 0.15b
Cotton	4	NN	0.07 ± 0.01a	0.59 ± 0.01b	0.67 ± 0.01b	483.88 ± 50.97b	2.41 ± 0.30a
		LN	0.08 ± 0.02a	0.75 ± 0.02a	0.83 ± 0.03a	679.72 ± 79.08a	1.18 ± 0.36b
	8	NN	0.13 ± 0.01a	0.71 ± 0.01a	0.84 ± 0.03a	179.14 ± 10.97a	2.37 ± 0.34a
		LN	0.12 ± 0.04a	0.67 ± 0.08a	0.79 ± 0.12a	74.87 ± 19.27b	0.32 ± 0.04b

*Different lowercase letters in same column mean significant differences between the control treatment and N deficiency on the same time in wheat and cotton according to the LSD test (p = 0.05).*

**FIGURE 3:**
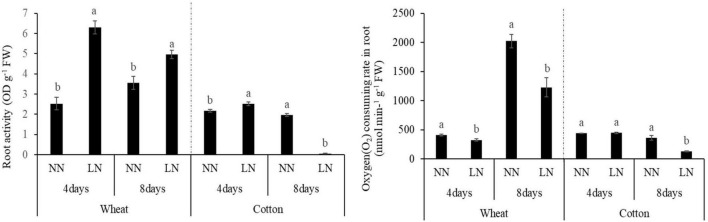
The effects of N deficiency on root physiological traits of wheat and cotton seedlings. Different lowercase letters above the bars mean significant differences between the control treatment and N deficiency on the same time in wheat and cotton according to the LSD test (*p* = 0.05).

The physiological function of roots was also significantly impacted by N deficiency, which was evidenced by the change of root activity and respiration (Figure 3). Compared with the controls, the root activities were significantly increased after 4 days of N deficiency treatments in both crops. However, after 8 days of treatments, the root activities were changed in an opposite direction with the value of wheat increased 39.31% and the value of cotton decreased 97.31% by compared with the control treatment. Under N deficiency, oxygen consuming rate in root of wheat was significantly decreased, by 21.19% on the 4th and 39.35% on 8th days than that of the control. While compared with the control, the root oxygen consuming rate was significantly decreased by 64.77% in cotton until the 8th days of N deficiency.

### Effects of N Deficiency on the Expression of miRNAs in Wheat and Cotton Roots

**FIGURE 4:**
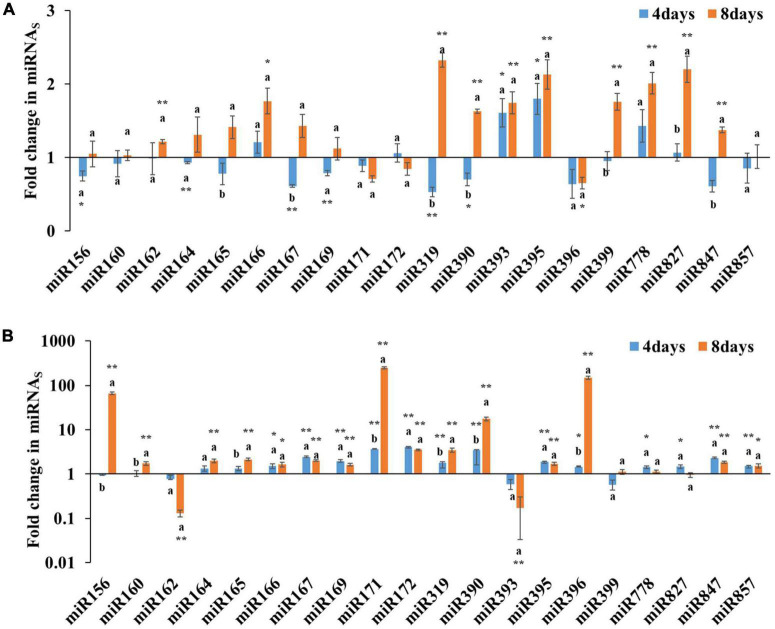
Expression fold changes of miRNAs after 4 and 8 days of N deficiency treatments in wheat **(A)** and cotton **(B)**. The different letters above the bars imply significant differences about expression of miRNA targets between the 4 and 8 days of N treatment according to the LSD test (*P* = 0.05). Highly significant difference between N deficiency and the controls was marked as ** to indicate a *p*-value < 0.01 and as * to indicate a *p*-value < 0.05.

N deficiency significantly altered the expression of miRNAs. Different miRNAs responded differently to N deficiency with a time- and crop-dependent manner (Figure 4). Even a same miRNA responded differently in different plant species at the same time point. On the 4th days of N deficiency treatment, the majority of tested miRNAs were inhibited in wheat except miR166, miR172, miR393, miR395, miR778, and miR827. Among all tested miRNAs in wheat, miR156, miR164, miR167, miR169, miR319, miR390, miR393, miR395 showed different expression levels after 4 days of N deficiency treatment. In contrast, most of the tested miRNAs in wheat, were up-regulated after 8 days of N deficiency treatment, except miR171, miR172, miR396. Among all tested miRNAs, the expressions of miR162, miR166, miR319, miR390, miR393, miR395, miR396, miR399, miR778, miR827, miR847 were significantly altered in wheat after 8 days of N deficiency, compared with the control. With the duration of N deficiency, the expressions of miR165, miR167, miR319, miR390, miR399, miR827, miR847 were significantly higher in wheat roots than that after 4 days of treatment (Figure 4A).

In cotton, except miR156, miR162, miR393, and miR399, the majority of tested miRNAs were up-regulated after 4 days of N deficiency treatment. Among the up-regulated miRNAs, miR172 showed the most up-regulated level by 4.01-folds. miR167, miR171, miR390, and miR847 were also up-regulated by at least 2-folds after 4 days of N deficiency treatment. After 4 days of treatments, except miR156, miR160, miR162, miR164, miR165, miR393, and miR399, the expressions of all tested miRNAs were significantly difference between the N deficiency treatment and the control. After 8 days of treatments, except miR162 and miR393, all tested miRNAs were up-regulated under N deficiency treatment. Among the up-regulated miRNAs, miR156, miR171, miR390, miR396 were induced by 66.28, 250.02, 17.57, and 149.74-folds after 8 days N deficiency treatments. In addition, except miR399, miR778, and miR827, the expression of all tested miRNAs had a significant difference by comparing with the control after 8 days of N deficiency treatments. With the duration of N deficiency treatment, the expressions of miR156, miR160, miR165, miR171, miR319, miR390, miR396 were significantly higher at 8 days than that at the 4th days of treatment (Figure 4B).

### Effects of N Deficiency on the Expression of Targeted mRNAs by miRNAs

**FIGURE 5:**
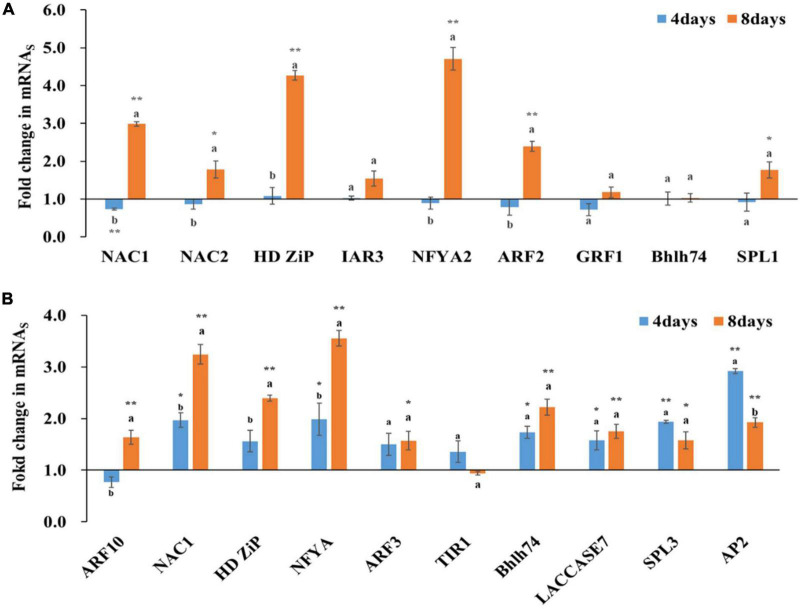
Expression fold changes of miRNA targets after 4 and 8 days of N deficiency treatments in wheat **(A)** and cotton **(B)**. The different letters above the bars imply significant differences about expression of miRNA targets between the 4 and 8 days of N treatment according to the LSD test (*P* = 0.05). Highly significant difference between N deficiency and the controls was marked as ** to indicate a *p*-value < 0.01 and as * to indicate a *p*-value < 0.05.

Not only the expression of miRNAs was affected by N levels and the treatment period, the expression of their targeted genes was also obviously affected, and different miRNA targets responded differently to N deficiency (Figure 5). In wheat, all tested miRNA targets were down-regulated after 4 days of N deficiency treatments, except HDZiP, IAR3, bHLH74. Among the tested miRNA targets, only NAC1 showed a significant difference by comparing with the control after 4 days of N deficiency treatment. After 8 days of treatments, all of tested miRNA targets were induced by N deficiency treatment; except IAR3, GRF1, bHLH74, their expressions were significantly higher in N deficiency treatment than that in the control. With the duration of the N deficiency, except IAR3, GRF1, bHLH74, and SPL1, the expressions of all tested miRNA targets had significant difference after 8 days of treatment compared with after 4 days of treatment (Figure 5A).

In cotton, except ARF10, all of tested miRNA targets were up-regulation after 4 days of N deficiency treatments. The expressions of NAC1, NFYA, Bhlh74, LACCASE7, SPL3, AP2 were significantly different between the treatment and the control after 4 days of N deficiency treatments. After 8 days of N deficiency treatments, except T1R1, all of tested miRNA targets were up-regulation, and had significant difference by comparing with the control. Among the up-regulated miRNA targets, the expression of NAC1 and NFYA were induced by more than 3-folds, the expression of HD ZiP and Bhlh74 were induced by more than 2-folds after 8 days of N deficiency treatment. Compared the gene expression between 4 and 8 days’ treatment, the expressions of ARF10, NAC1, HDZiP, NFYA, AP2 were significantly altered (Figure 5B).

### The Expression Relationship Between miRNAs and Their Targets Under N Deficiency

**FIGURE 6:**
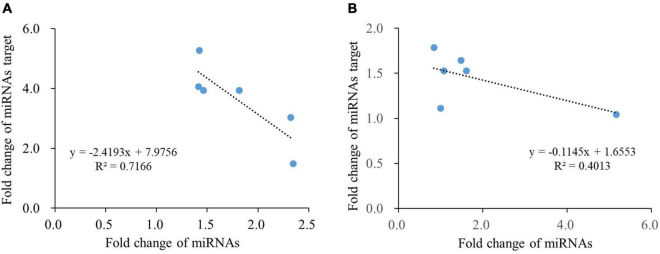
Relationship between miRNAs and their targets under N deficiency in roots of wheat **(A)** and cotton **(B)**.

Generally speaking, the expression of miRNAs had a negative correlation with the expression of their targeted genes, because miRNAs regulated plant growth and development by inhibiting protein translation or degradation of mRNAs. In a real-world scenario, there was a complicated expression correlation between miRNAs and their targeted mRNAs. For example, when secondary reactions or DNA methylation working, the negative expression correlation between miRNAs and their targets was disappeared. In order to reveal the correlation between miRNAs and their targets, a linear equation was modeled between the fold changes of 6 tested miRNAs and their targets of both crops from 4 to 8 days under N deficiency. As shown in Figure 6, negative liner relationships were shown with the equation: *y* = -2.4193X + 7.9756 (*R*^2^ = 0.7166) in wheat, and the equation: *y* = -0.1145X + 1.6553 (*R*^2^ = 0.4013) in cotton, which indicates a reverse expression correlation existed between the tested miRNAs and their targeted mRNAs.

## Discussion

### N Deficiency Differentially Affected Morpho-Physiological Characteristics of Shoot and Root in Wheat and Cotton Seedlings

N as one major type of macro-elements involved in a series of physiological and biochemical processes in plant, such as gene expression, metabolism, plant growth, and yield formation (Nava et al., 2007; Cunha et al., 2014). Therefore, N deficiency directly limited the plant growth reflected by change of the plant dry weight. Photosynthesis is the key process of plant metabolism, assimilating CO_2_ and releasing O_2_ at the same time (Kalaji et al., 2014). The chlorophyll a fluorescence parameter was well correlated with photosynthesis (Heerden et al., 2003; Ripley et al., 2004). PI_ABS_ was decreased notably with the duration of N deficiency, while only the chlorophyll b and total chlorophyll content were significantly increased in cotton leaves after 4 days of N deficiency treatment; the chlorophyll a and total chlorophyll content were significantly decreased in wheat leaves after 8 days of N deficiency by comparing with the control (Table 2). The O_2_ consuming rate in leaves reflected the respiration. When the environment was not favorable, the plant would increase its respiration rate to produce more energy to counter the adverse conditions (Bazzaz, 1979). After 4 days of N deficiency treatments, the O_2_ consuming rates of both crop leaves had an opposite response, with a significantly decline in wheat and significantly ascent in cotton. However, the O_2_ consuming rates of wheat and cotton leaves were significantly decreased with different degrees after 8 days of N deficiency treatments.

Plant root system was the important organ for absorbing water and nutrient, which can change its three-dimensional deployment to respond the water and nutrient availability and distribution in the soil (Linkohr et al., 2002). Previous studies demonstrated that root systems could rapidly extend to the deep soil for capturing more water and N when facing an abiotic stress condition, such as leaking of water and N (Lynch, 2013; Trachsel et al., 2013; Lynch and Wojciechowski, 2015). Therefore, correlations between root morphological characteristics and N supply were further studied in this experiment. Although N deficiency did not significantly impact the root biomass, an obvious effect was observed on the root morphological traits of wheat and cotton. After 8 days of N treatment, the root morphological traits of both corps had an opposite response, with the total root length, root surface area, and root volume significant reduced in wheat and increased in cotton, while lateral root density significantly increased in wheat and reduced in cotton by compared with the controls. In addition, the seminal root length in wheat and lateral root length in cotton both obviously increased after 8 days of N treatment (Table 1 and Figure 2). The results indicated that morphological adaptation mechanism of plant root to N deficiency varied in monocot wheat and dicot cotton.

Root respiration was a major trait reflecting the physiological metabolic capacity of root (Ryan et al., 1996; Wells and Eissenstat, 2002), which involved in the resource acquisition strategies of root (Han and Zhu, 2021) and provided the driving force for root growth (Lambers et al., 2008). Jia et al. (2010) proposed that N fertilization increased fine root respiration rates in *Larix gmelinii* (larch) and *Fraxinus mandshurica* (ash). In our study, the O_2_ consuming rate was significantly decreased in both wheat and cotton roots after 8 days of N deficiency treatment, which might be caused by the soluble sugars deficit (Saglio and Pradet, 1980). High root respiration usually accompanied with high root activity (Fontana et al., 2020). A positive relationship of root respiration and vitality was observed under K^+^ deficiency in both wheat and cotton (Fontana et al., 2020; Thornburg et al., 2020). In this study, root activity and the O_2_ consuming rate showed an opposite trend in wheat roots; while root activity and the O_2_ consuming rate had a positive relationship in cotton roots after 8 days of N treatment (Figure 3). The possible reason may be that root activity reflected the strength of the whole root metabolism, not only the respiration but also the absorbing ability, biosynthesis, and oxidation activity (Liu X. et al., 2009). Root vitality indicated the activity of root and was the most importance to recognize the active fine root biomass that was available for uptaking water and nutrients (Rewald and Meinen, 2013). Combined with root morphological results, it was possibly deduced that the monocot wheat adapted the N deficiency by allocating more energy to nutrient uptake, and the dicot cotton adapted the N deficiency by allocating more energy to root extension.

### Synergistically Regulation of miRNAs and Their Targets for Root Growth Under N Deficiency in Wheat and Cotton

miRNAs participated in almost all biological, metabolic progresses and stress response in plant (Liu et al., 2021). Therefore, it had become an important modern genetic manipulation technique to increase crop yield and tolerance to abiotic stresses. Dicer-like (DCL) proteins regulated by miR162 in *Arabidopsis* were key components of small RNA biogenesis (Moissiard et al., 2007; Mlotshwa et al., 2008; Liu Q. P. et al., 2009). DCL1 and DCL3 redundantly acted to expedite *Arabidopsis* flowering (Schmitz et al., 2007). By targeting the plant-specific transcription factors TCP (for TEOSINTEBRANCHED/CYCLOIDEA/PCF), the conserved miR319 participated in many important progresses of plant development, including the regulation of cell division, expansion, and differentiation during leaf development, mitochondrial biogenesis, floral development, and leaf senescence (Palatnik et al., 2003; Schommer et al., 2008; Martín-Trillo and Cubas, 2010). Among plant miRNA genes, 22 families were founded conserved between the monocots and the dicots (Sorin et al., 2014). Therefore, the miRNA-mediated mechanism of root growth and development in monocots and dicots under N deficiency was investigated in this study. In our previous study, we found that, except miR399, miR778, miR857, ARF2, and GRF, almost all tested miRNAs and their targets were up-regulated with an inhibition of total root growth, primary root growth and lateral root formation in peanut after 8 days of N deficiency (Li L. et al., 2021). In this study, we found that miR167, miR319, miR390, miR827, and miR847 were also induced by more than 2-fold in wheat after 8 days of N deficiency by comparing with their expression levels at 4 days of N deficiency; we also observed that N deficiency inhibited the total root growth but promoted the seminal roots growth and lateral root formation to tolerate N deficit. In cotton, miR156 and miR396 were up-regulated by more than 2-folds but miR162 and miR393 were inhibited at least 2-folds by N deficiency after 8 days of treatment. We also observed that miR167, miR171, miR172, miR390 were up-regulated by N deficiency, which enhanced the total root and primary growth while inhibited the lateral root formation to adapt to N deficit in cotton (Table 1 and Figures 2, 4). Thus, the monocots and the dicots had different root regulatory mechanisms of miRNAs to N deficiency, and miR167 participated the response to N deficiency in both crops. Li L. et al. (2021) reported that miR167 participated in the regulation of lateral root growth under N deficiency and K deficiency treatment in peanut. With the duration of K deficiency, miR167 was down-regulated by 2-folds to regulate the root growth in wheat (Thornburg et al., 2020) and cotton (Fontana et al., 2020). Therefore, miR167 was involved in regulating the responses of crops to K deficiency and N deficiency, but significantly down-regulated under K deficiency conditions and significantly up-regulated in N deficiency. In *Arabidopsis thaliana* and *Oryza sativa*, miR827 regulated phosphate (Pi) transport and storage which played major roles in affecting plant growth by targeting two different types of SPX (SYG1/PHO81/XPR1)-domain-containing genes, NITROGEN LIMITATION ADAPTATION (NLA) and PHOSPHATE TRANSPORTER 5 (PHT5) (Lin et al., 2010; Azevedo and Saiardi, 2017). Therefore, the phosphate homeostasis was accelerated in wheat by upregulating miR827 under N deficiency, which may promote seminal root growth and lateral root development.

Auxin controlled and regulated root development in different stage to adapt the environment (Lu et al., 2018). At several major stages of root development, genes involved in auxin signaling pathway were proved to be the targets of certain miRNAs (Jodder, 2020). Auxin response factors (ARFs) which were a family of transcription factors (TFs) (Yang et al., 2006), could promote and regulate auxin responsive genes expression by binding to auxin response elements (AuxRE) (Guilfoyle and Hagen, 2007). Plants with reduced levels of ARF10 and ARF16 targeted by miR160c had shorter and agravitropic roots with enlarged tumor-like apex (Khan et al., 2011). miR167 functioned *via* targeted the IAA-Ala resistant3 (IAR3) genes and ARFs, such as ARF6, ARF8, ARF12, ARF17, ARF25 (Liu et al., 2021), played essential roles in both reproductive processes and root development in plant (Meng et al., 2010). Upregulated miR390 produced the ta-siRNA (trans-acting short-interfering RNAs) by targeting non-coding TAS3 precursor RNA, then cleaved the ARFs (ARF2/3/4) to promote the lateral root growth (Williams et al., 2005; Cabrera et al., 2016). In addition, miR393 also participated in auxin signaling pathway by targeting the auxin receptors, auxin F-box protein 1, 2, 3 (AFB1/AFB2/AFB3), and transport inhibitor response-1(TIR1) (Jodder, 2020), therefore functioned in leaf and root development (Chen et al., 2010; Si-Ammour et al., 2011; Chen et al., 2012). Plants with low expression of miR164 produced more lateral roots by targeting the NAC family (Guo et al., 2005), while NAC1 acted as the downstream of TIR1 to transmit the auxin signals (Xie et al., 2000). miR847 upregulated auxin signaling to mediate lateral organ development by cleaving the auxin/indole acetic acid (Aux/IAA) repressor-encoding gene IAA28 in *Arabidopsis* (Wang and Guo, 2015). In this study, the expression levels of miR160, miR164, miR167, miR390, miR393, and miR847 were up-regulated in wheat after 8 days of N deficiency treatments (Figure 4A). Therefore, the increased lateral root density may be the subtle balance of activator and repressor ARF transcripts in wheat under N deficiency. In cotton, the expression levels of miR160, miR164, miR167, miR390, miR393, and miR847 were up-regulated after 8 days of N deficiency treatments (Figure 4B). The decreased lateral root density may also be the subtle balance of activator and repressor ARF transcripts in cotton under N deficiency. Therefore, the networks regulated by miRNAs exited an interaction during root development, but the monocots and the dicots executed different root regulatory mechanisms of miRNAs.

**FIGURE 7:**
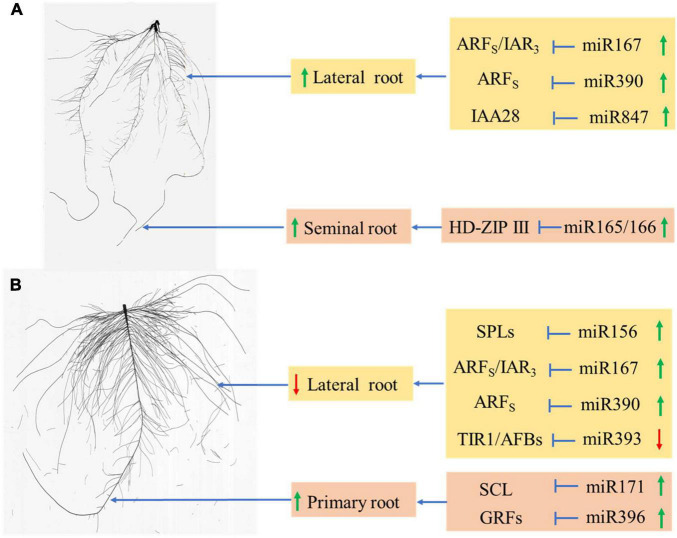
The potential miRNAs-mediated mechanism for root growth in wheat **(A)** and cotton **(B)** by controlling the expression of protein-coding genes under N deficiency. The green arrow and red arrow represent the upregulated and downregulated expression of miRNAs, respectively.

Among the targets of miRNAs, approximately 66% were TFs (Tang and Chu, 2017). For example, the well-conserved miR156 regulated plant development by targeting SPL TFs (Schwab et al., 2005; Axtell and Bowman, 2008); one group of SPL genes including SPL3, SPL9, and SPL10 were participated in the inhibition of lateral root growth (Yu et al., 2015). Previously research found SPL9, SPL10 promoted the expression of miR172 (Wu et al., 2009), whose family members played functional specificity in regulating stem elongation, meristem size, shoot branching, trichome initiation, and floral competence (Lian et al., 2021). Yu et al. (2015) unraveled the role of miR156/SPLs modules in lateral root formation in *Arabidopsis*. In addition, plants with high expression of miR156 reduced meristem size, resulting in shorter primary root (Barrera et al., 2020). In this study, the expression level of miR156 had no significant change in wheat after 8 days of N deficiency by compared with the control treatment (Figure 4A). The class III HOMEODOMAIN-LEUCINE ZIPPER (HD-ZIP III) was negatively restricted by miR165/166, and overexpression of miR165/166 not only regulated the root radial patterning but also increased primary root length by regulating the meristematic activity (Carlsbecker et al., 2010; Singh et al., 2014). In this research, the expression levels of miR165 and miR166 were both up-regulated, but less than 2-fold on the 8th day of N deficiency by compared with the control treatment. The seminal root length in wheat increased but had no significant difference by comparing with the control treatment on the 8th day of N deficiency (Table 1 and Figures 2, 4A). Thus, we concluded that the miR165/166-HD-ZIP III module participated in the seminal root growth in wheat (Figure 7A). In cotton, the expression level of miR156 was significantly upregulated with longer primary root, lower lateral root density after 8 days of N deficiency (Table 1 and Figures 2, 4B). So, we could know the overexpression of miR156 inhibited the lateral root formation in cotton (Figure 7B). miR171 was proved to regulate primary root elongation through manipulating the quiescent centre and by targeting SCARECROW-LIKE6-II (SCL6-II), SCL6-III and SCL6-IV in *Arabidopsis* under N starvation (Wang et al., 2010; Zhou et al., 2015; Yan et al., 2022). In *Medicago truncatula* and *Arabidopsis*, miR396 targeting GRFs impeded primary root growth by regulating cell division (Bazin et al., 2013; Rodriguez et al., 2015; Yan et al., 2022). In this manuscript, the expression levels of miR171, miR396 had significantly upregulated above 2-fold on the 8th day of N deficiency by comparing with the 4th day of N deficiency in cotton (Figure 4B). Therefore, the promotion of primary root development may be related to miR171 and miR396 regulation models (Figure 7B).

## Conclusion

Under N deficiency treatment, the root dry biomass had no significant change, but the total root growth was inhibited in wheat and promoted in cotton. In addition, the lateral root density was increased in wheat and declined in cotton, while seminal root length in wheat and primary root length were both increased after 8 days of N treatments. Furthermore, the miRNA-mediated mechanisms were different under N deficiency in monocot and dicot, In wheat, overexpression of miR167, miR319, miR390, miR827, miR847, and miR165/166 coacted to inhibit the total root growth but promote the seminal roots growth and lateral root formation to tolerate N deficit. In cotton, overexpression of miR156, miR167, miR171, miR172, miR390, and miR396 and reduced expression of miR162 and miR393 combinedly worked to enhance the total root and primary growth and to inhibit the lateral root formation to adapt to N deficit.

## Data Availability Statement

The original contributions presented in this study are included in the article/supplementary material, further inquiries can be directed to the corresponding authors.

## Author Contributions

HX: analyzing the data and writing – review and editing the original draft. JL: performing experiments. CP, SO, and WL: data curation and investigation. QL and GW: investigation and validation. LL: investigation and analyzing the data. ZZ, XP, and BZ: formal analysis and writing – review and revising the manuscript. All authors contributed to the article and approved the submitted version.

## Conflict of Interest

The authors declare that the research was conducted in the absence of any commercial or financial relationships that could be construed as a potential conflict of interest.

## Publisher’s Note

All claims expressed in this article are solely those of the authors and do not necessarily represent those of their affiliated organizations, or those of the publisher, the editors and the reviewers. Any product that may be evaluated in this article, or claim that may be made by its manufacturer, is not guaranteed or endorsed by the publisher.

## References

[B1] Asseng S. Ritchie J. T. Smucker A. J. M. Robertson M. J. (1998). Root growth and water uptake during water deficit and recovering in wheat. *Plant Soil.* 201 265–273. 10.1023/A:1004317523264

[B2] Axtell M. J. Bowman J. L. (2008). Evolution of plant microRNAs and their targets. *Trends Plant Sci.* 13 343–349. 10.1016/j.tplants.2008.03.009 18502167

[B3] Azevedo C. Saiardi A. (2017). Eukaryotic phosphate homeostasis: the inositol pyrophosphate perspective. *Trends Biochem. Sci.* 42 219–231. 10.1016/j.tibs.2016.10.008 27876550

[B4] Barrera C. Rocha G. H. B. Polverari L. Brito D. A. P. Nogueira F. T. S. (2020). miR156-targeted SPL10 controls *Arabidopsis* root meristem activity and root-derived de novo shoot regeneration via cytokinin responses. *J. Exp. Bot.* 71 934–950. 10.1093/jxb/erz475 31642910

[B5] Bartel D. P. (2009). MicroRNAs: target recognition and regulatory functions. *Cell* 136 215–233. 10.1016/j.cell.2009.01.002 19167326 PMC3794896

[B6] Bazin J. Khan G. A. Combier J. P. Bustos-Sanmamed P. Debernardi J. M. Rodriguez R. (2013). miR396 affects mycorrhization and root meristem activity in the legume *Medicago truncatula*. *Plant J.* 74 920–934. 10.1111/tpj.12178 23566016

[B7] Bazzaz F. A. (1979). The physiological ecology of plant succession. *Annu. Rev. Ecol. Syst.* 10 351–371. 10.1146/annurev.es.10.110179.002031

[B8] Bellini C. Pacurar D. I. Perrone I. (2014). Adventitious roots and lateral roots: similarities and differences. *Annu. Rev. Plant Biol.* 65 639–666. 10.1146/annurev-arplant-050213-035645 24555710

[B9] Cabrera J. Barcala M. Garcia A. Rio-Machin A. Medina C. Jaubert-Possamai S. (2016). Differentially expressed small RNAs in Arabidopsis galls formed by *Meloidogyne javanica*: a functional role for miR390 and its TAS3-derived tasiRNAs. *New Phytol.* 209 1625–1640. 10.1111/nph.13735 26542733

[B10] Carlsbecker A. Lee J. Roberts C. J. Dettmer J. Lehesranta S. Zhou J. (2010). Cell signalling by microRNA165/6 directs gene dose-dependent root cell fate. *Nature* 465 316–321. 10.1038/nature08977 20410882 PMC2967782

[B11] Carpenter S. Caraco N. Correll D. Howarth R. Sharpley A. Smith V. (1998). Nonpoint pollution of surface waters with phosphorus and nitrogen. *Ecol. Appl.* 8 559–568. 10.1890/1051-0761(1998)008

[B12] Chen H. Li Z. F. Xiong L. M. (2012). A plant microRNA regulates the adaptation of roots to drought stress. *FEBS Lett.* 586 1742–1747. 10.1016/j.febslet.2012.05.013 22613571

[B13] Chen Z. H. Sun Y. Z. Yang Y. J. Xu X. H. Wang J. H. Han N. (2010). Regulation of auxin response by miR393-targeted transport inhibitor response protein 1 is involved in normal development in *Arabidopsis*. *Plant Mol. Biol.* 2010 619–629. 10.1007/s11103-011-9838-1 22042293

[B14] Chiou T. J. (2010). The role of microRNAs in sensing nutrient stress. *Plant Cell Environ.* 30 323–332. 10.1111/j.1365-3040.2007.01643.x 17263777

[B15] Chuck G. Cigan A. M. Saeteurn K. Hake S. (2007). The heterochronic maize mutant corngrass1 results from overexpression of a tandem microRNA. *Nat. Genet.* 39 544–549. 10.1038/ng2001 17369828

[B16] Cui H. Levesque M. P. Vernoux T. Jung J. W. Paquette A. J. Gallagher K. L. (2007). An evolutionarily conserved mechanism delimiting SHR movement defines a single layer of endodermis in plants. *Science* 316 421–425. 10.1126/science.1139531 17446396

[B17] Cunha M. Cavalcante ÍH. L. Mancin A. C. Albano F. G. Marques A. (2014). Impact of humic substances and nitrogen fertilising on the fruit quality and yield of custard apple. *Acta Sci. Agron.* 37 211–218.

[B18] Engineer C. B. Kranz R. G. (2007). Reciprocal leaf and root expression of atamt1.1 and root architectural changes in response to nitrogen starvation. *Plant Physiol.* 143 236–250. 10.1104/pp.106.088500 17085512 PMC1761975

[B19] Feng S. J. Xu Y. M. Guo C. K. Zheng J. R. Zhou B. Y. Zhang Y. T. (2016). Modulation of miR156 to identify traits associated with vegetative phase change in tobacco (*Nicotiana tabacum*). *J. Exp. Bot.* 67 1493–1504. 10.1093/jxb/erv551 26763975

[B20] Fontana J. E. Wang G. Sun R. Xue H. Li Q. Liu J. (2020). Impact of potassium deficiency on cotton growth, development and potential microRNA-mediated mechanism. *Plant Physiol. Biochem.* 153 72–80. 10.1016/j.plaphy.2020.05.006 32480238

[B21] Fuentes S. I. Allen D. J. Adriana O. L. Georgina H. (2001). Over-expression of cytosolic glutamine synthetase increases photosynthesis and growth at low nitrogen concentrations. *J. Exp. Bot.* 52 1071–1081. 10.1093/jexbot/52.358.1071 11432923

[B22] Gao J. Wang F. Hu H. Jiang S. Muhammad A. Shao Y. (2018). Improved leaf nitrogen reutilisation and rubisco activation under short-term nitrogen-deficient conditions promotes photosynthesis in winter wheat (*Triticum aestivum* L.) at the seedling stage. *Funct. Plant Biol.* 45 840. 10.1071/FP17232 32291066

[B23] Gao K. U. N. Chen F. Yuan L. Zhang F. Mi G. (2015). A comprehensive analysis of root morphological changes and nitrogen allocation in maize in response to low nitrogen stress. *Plant Cell Environ.* 38 740–750. 10.1111/pce.12439 25159094

[B24] Guilfoyle T. J. Hagen G. (2007). Auxin response factors.curr. *Opin. Plant Biol.* 10 453–460. 10.1016/j.pbi.2007.08.014 17900969

[B25] Guo H. S. Xie Q. Fei J. F. Chua N. H. (2005). MicroRNA directs mRNA cleavage of the transcription factor NAC1 to downregulate auxin signals for *Arabidopsis* lateral root development. *Plant Cell* 17 1376–1386. 10.1105/tpc.105.030841 15829603 PMC1091761

[B26] Hakoomat A. H. Shakeel A. Ahmad N. Shahzad R. Atif H. (2014). Efficacy of different techniques of nitrogen application on American cotton undersemi-arid conditions. *J. Food, Agric. Environ.* 12 157–160.

[B27] Han M. G. Zhu B. (2021). Linking root respiration to chemistry and morphology across species. *Global Change Biol.* 27 190–201. 10.1111/gcb.15391 33058350

[B28] Heerden P. D. R. Tsimilli-Michael M. Kruger G. H. J. Strasser R. J. (2003). Dark chilling effects on soybean genotypes during vegetative development: parallel studies of CO2 assimilation, chlorophyll a fluorescence kinetics O-J-I-P and nitrogen fixation. *Physiol. Plant.* 117 476–491. 10.1034/j.1399-3054.2003.00056.x 12675738

[B29] Hochholdinger F. Zimmermann R. (2008). Conserved and diverse mechanisms in root development. *Curr. Opin. Plant Biol.* 11 70–74. 10.1016/j.pbi.2007.10.002 18006363

[B30] Jia S. Wang Z. Li X. Yue S. Zhang X. Liang A. (2010). N fertilization affects on soil respiration, microbial biomass and root respiration in *Larix gmelinii* and *Fraxinus mandshurica* plantations in China. *Plant Soil* 333 325–336. 10.1007/s11104-010-0348-8

[B31] Jiang K. Meng Y. L. Feldman L. J. (2003). Quiescent center formation in maize roots is associated with an auxin-regulated oxidizing environment. *Development* 130 1429–1438. 10.1242/dev.00359 12588857

[B32] Jodder J. (2020). miRNA-mediated regulation of auxin signaling pathway during plant development and stress responses. *J. Biosci.* 45:91. 10.1007/s12038-020-00062-1 32713854

[B33] Kalaji H. M. Oukarroum A. Alexandrov V. (2014). Identification of nutrient deficiency in maize and tomato plants by in vivo chlorophyll a fluorescence measurements. *Plant Physiol. Bioch.* 81 16–25. 10.1016/j.plaphy.2014.03.029 24811616

[B34] Khan G. A. Declerck M. Sorin C. Hartmann C. Crespi M. Lelandais-Brière C. (2011). MicroRNAs as regulators of root development and architecture. *Plant Mol. Biol.* 77 47–58. 10.1007/s11103-011-9793-x 21607657

[B35] Lambers H. Chapin F. Pons T. (2008). *Plant Physiological Ecology*, 2nd Edn. New York: Springer.

[B36] Li C. Zhang B. H. (2016). MicroRNAs in control of plant development. *J. Cell. Physiol.* 2016 303–313. 10.1002/jcp.25125 26248304

[B37] Li T. Lian H. Li H. Xu Y. Zhang H. (2021). HY5 regulates light-responsive transcription of microRNA163 to promote primary root elongation in *Arabidopsis* seedlings. *J. Integr. Plant Biol.* 63 14. 10.1111/jipb.13099 33860639

[B38] Li L. Li Q. Davis K. E. Patterson C. Oo S. Liu W. (2021). Response of root growth and development to nitrogen and potassium deficiency as well as microRNA-mediated mechanism in peanut (*Arachis hypogaea* L.). *Front. Plant Sci.* 12:695234. 10.3389/fpls.2021.695234 34178008 PMC8231928

[B39] Lian H. Wang L. Ma N. Zhou C. M. Han L. Zhang T. Q. (2021). Redundant and specific roles of individual MIR172 genes in plant development. *PLoS Biol.* 19:3001044. 10.1371/journal.pbio.3001044 33529193 PMC7853526

[B40] Lin S. I. Santi C. Jobet E. Lacut E. El Kholti N. Karlowski W. M. (2010). Complex regulation of two target genes encoding SPX-MFS proteins by rice miR827 in response to phosphate starvation. *Plant Cell Physiol.* 51 2119–2131. 10.1093/pcp/pcq170 21062869

[B41] Linkohr B. I. Williamson L. C. Fitter A. H. Leyser H. M. O. (2002). Nitrate and phosphate availability and distribution have different effects on root system architecture of *Arabidopsis*. *Plant J.* 29 751–760. 10.1046/j.1365-313x.2002.01251.x 12148533

[B42] Liu Q. P. Feng Y. Zhu Z. J. (2009). Dicer-like (DCL) proteins in plants. *Funct. Integr. Genomic.* 9 277–286. 10.1007/s10142-009-0111-5 19221817

[B43] Liu X. Huang S. Xie H. (2021). Advances in the regulation of plant development and stress response by miR167. *Front. Biosci. Land.* 26:655–665. 10.52586/4974 34590474

[B44] Liu X. Zhang F. Yang Q. Li Z. (2009). Response of chlorophyll, proline and root activity of maize to regulated deficit irrigation and N fertilization treatment. *Acta Agric. Boreali Sin.* 24 106–111.

[B45] Lu Y. Feng Z. Liu X. Bian L. Xie H. Zhang C. (2018). MiR393 and miR390 synergistically regulate lateral root growth in rice under different conditions. *BMC Plant Biol.* 18:261. 10.1186/s12870-018-1488-x 30373525 PMC6206659

[B46] Lynch J. P. (2013). Steep, cheap and deep: an ideotype to optimize water and N acquisition by maize root systems. *Ann. Bot.* 112 347–357. 10.1093/aob/mcs293 23328767 PMC3698384

[B47] Lynch J. P. Wojciechowski T. (2015). Opportunities and challenges in the subsoil: pathways to deeper rooted crops. *J. Exp. Bot.* 66 2199–2210. 10.1093/jxb/eru508 25582451 PMC4986715

[B48] Martín-Trillo M. Cubas P. (2010). TCP genes: a family snapshot ten years later. *Trends Plant Sci.* 15 31–39. 10.1016/j.tplants.2009.11.003 19963426

[B49] Meng Y. Ma X. Chen D. Wu P. Chen M. (2010). MicroRNA-mediated signaling involved in plant root development. *Biochem. Biophys. Res. Commun.* 393 345–349. 10.1016/j.bbrc.2010.01.129 20138828

[B50] Mlotshwa S. Pruss G. J. Peragine A. Endres M. W. Li J. Chen X. (2008). DICER-LIKE2 plays a primary role in transitive silencing of transgenes in *Arabidopsis*. *PLoS One* 3:e1755. 10.1371/journal.pone.0001755 18335032 PMC2262140

[B51] Moissiard G. Parizotto E. A. Himber C. Voinnet O. (2007). Transitivity in *Arabidopsis* can be primed, requires the redundant action of the antiviral dicer-like 4 and dicer-like 2, and is compromised by viral-encoded suppressor proteins. *RNA* 22 1268–1278. 10.1261/rna.541307 17592042 PMC1924903

[B52] Nava G. Dechen A. R. Nachtigall G. R. (2007). Nitrogen and potassium fertilization affect apple fruit quality in southern Brazil. *Commun. Soil Sci. Plan.* 39 96–107. 10.1080/00103620701759038

[B53] Osmont K. S. Sibout R. Hardtke C. S. (2007). Hidden branches: developments in root system architecture. *Annu. Rev. Plant Biol.* 58 93–113. 10.1146/annurev.arplant.58.032806.104006 17177637

[B54] Palatnik J. F. Allen E. Wu X. Schommer C. Weigel D. (2003). Control of leaf morphogenesis by miRNAs. *Nature* 425 257–263. 10.1038/nature01958 12931144

[B55] Rewald B. Meinen C. (2013). Plant roots and spectroscopic methods – analyzing species, biomass and vitality. *Front. Plant Sci.* 4:393. 10.3389/fpls.2013.00393 24130565 PMC3793172

[B56] Richter J. Roelcke M. (2000). The N-cycle as determined by intensive agriculture – examples from central Europe and China. *Nutr. Cycl. Agroecosys.* 57 33–46. 10.1023/A:1009802225307

[B57] Ripley B. S. Redfern S. P. Dames J. F. (2004). Quantification of the photosynthetic performance of phosphorus-deficient sorghum by means of chlorophyll-a fluorescence kinetics. *Soc. Afr. J. Sci.* 100 615–618. https://hdl.handle.net/10520/EJC96177

[B58] Rodriguez R. E. Ercoli M. F. Debernardi J. M. Breakfield N. W. Mecchia M. A. Sabatini M. (2015). MicroRNA miR396 regulates the switch between stem cells and transit-amplifying cells in *Arabidopsis* roots. *Plant Cell* 27 3354–3366. 10.1105/tpc.15.00452 26645252 PMC4707450

[B59] Ryan M. Hubbard R. Pongracic S. Raison R. McMurtrie R. (1996). Foliage, fine-root, woody-tissue and stand respiration in pinus radiata in relation to nitrogen status. *Tree Physiol.* 16 333–343. 10.1093/treephys/16.3.333 14871734

[B60] Saglio P. H. Pradet A. (1980). Soluble sugars, respiration, and energy charge during aging of excised maize root tips. *Plant Physiol.* 66 516–519. 10.1104/pp.66.3.516 16661466 PMC440664

[B61] Schiefelbein J. W. Masucci J. D. Wang H. (1997). Building a root: the control of patterning and morphogenesis during root development. *Plant Cell* 9 1089–1098. 10.1105/tpc.9.7.1089 9254932 PMC156982

[B62] Schmitz R. J. Hong L. Fitzpatrick K. E. Amasino R. M. (2007). DICER-LIKE 1 and DICER-LIKE 3 redundantly act to promote flowering via repression of FLOWERING LOCUS C in *Arabidopsis thaliana*. *Genetics* 176 1359–1362. 10.1534/genetics.107.070649 17579240 PMC1894598

[B63] Schommer C. Palatnik J. F. Aggarwal P. Chételat A. Cubas P. Farmer E. E. (2008). Control of jasmonate biosynthesis and senescence by miR319 targets. *PLoS Biol.* 6:e230. 10.1371/journal.pbio.0060230 18816164 PMC2553836

[B64] Schwab R. Palatnik J. F. Riester M. Schommer C. Schmid M. Weigel D. (2005). Specific effects of microRNAs on the plant transcriptome. *Dev. Cell* 8 517–527. 10.1016/j.devcel.2005.01.018 15809034

[B65] Shang L. Cai S. Ma L. Wang Y. Abduweli A. Wang M. (2015). Seedling root QTLs analysis on dynamic development and upon nitrogen deficiency stress in upland cotton. *Euphytica* 207 645–663. https://hdl.handle.net/10520/EJC96177

[B66] Si-Ammour A. Windels D. Arn-Bouldoires E. Kutter C. Ailhas J. Meins F. (2011). miR393 and secondary siRNAs regulate expression of the TIR1/AFB2 auxin receptor clade and auxin-related development of *Arabidopsis* leaves. *Plant Physiol.* 157 683–691. 10.1104/pp.111.180083 21828251 PMC3192580

[B67] Singh A. Singh S. Panigrahi K. C. S. Reski R. Sarkar A. K. (2014). Balanced activity of microRNA166/165 and its target transcripts from the class III homeodomain-leucine zipper family regulates root growth in *Arabidopsis thaliana*. *Plant Cell Rep.* 33 945–953. 10.1007/s00299-014-1573-z 24504657

[B68] Sorin C. Declerck M. Christ A. Blein T. Ma L. Lelandais-Briere C. (2014). A miR169 isoform regulates specific NF-YA targets and root architecture in *Arabidopsis*. *New Phytol.* 202 1197–1211. 10.1111/nph.12735 24533947

[B69] Stulen I. (1998). Impact of gaseous nitrogen deposition on plant functioning. *New Phytol.* 139 61–70. 10.1046/j.1469-8137.1998.00179.x

[B70] Sun C. H. Yu J. Q. Hu D. G. (2017). Nitrate: a crucial signal during lateral roots development. *Front. Plant Sci.* 8:485. 10.3389/fpls.2017.00485 28421105 PMC5379155

[B71] Tang J. Chu C. (2017). MicroRNAs in crop improvement: fine-tuners for complex traits. *Nat. Plants* 3:17077. 10.1038/nplants.2017.77 28665396

[B72] Thornburg T. E. Liu J. Li Q. Xue H. Wang G. Li L. (2020). Potassium deficiency significantly affected plant growth and development as well as microRNA-mediated mechanism in wheat (*Triticum aestivum* L.). *Front. Plant Sci.* 11:1219. 10.3389/fpls.2020.01219 32922417 PMC7456879

[B73] Tilman D. (1999). Global environmental impacts of agricultural expansion: the need for sustainable and efficient practices. *Proc. Natl. Acad. Sci. U.S.A.* 96 5995–6000. 10.1073/pnas.96.11.5995 10339530 PMC34218

[B74] Tiwar J. K. Buckseth T. Zinta R. Saraswati A. Singh R. K. Rawat S. (2020). Genome-wide identification and characterization of microRNAs by small RNA sequencing for low nitrogen stress in potato. *PLoS One* 15:e0233076. 10.1371/journal.pone.0233076 32428011 PMC7237020

[B75] Trachsel S. Kaeppler S. M. Brown K. M. Lynch J. P. (2013). Maize root growth angles become steeper under low N conditions. *Field Crops Res.* 140 18–31. 10.1016/j.fcr.2012.09.010

[B76] Vidal E. A. Araus V. Lu C. Parry G. Green P. J. Coruzzi G. M. (2010). Nitrate-responsive miR393/AFB3 regulatory module controls root system architecture in *Arabidopsis thaliana*. *Proc. Natl. Acad. Sci. U.S.A.* 107 4477–4482. 10.1073/pnas.0909571107 20142497 PMC2840086

[B77] Voinnet O. (2009). Origin, biogenesis, and activity of plant microRNAs. *Cell* 136 669–687. 10.1016/j.cell.2009.01.046 19239888

[B78] Wang J. J. Guo H. S. (2015). Cleavage of INDOLE-3-ACETIC ACID INDUCIBLE28 mRNA by microRNA847 upregulates auxin signaling to modulate cell proliferation and lateral organ growth in *Arabidopsis*. *Plant Cell* 27 574–590. 10.1105/tpc.15.00101 25794935 PMC4558675

[B79] Wang L. Mai Y. X. Zhang Y. C. Luo Q. Yang H. Q. (2010). MicroRNA171c-targeted SCL6-II, SCL6-III, and SCL6-IV genes regulate shoot branching in *Arabidopsis*. *Mol. Plant* 3 794–806. 10.1093/mp/ssq042 20720155

[B80] Wang M. Sun R. Li C. Wang Q. Zhang B. (2017). MicroRNA expression profiles during cotton (*Gossypium hirsutum* L) fiber early development. *Sci. Rep. Uk* 7:44454. 10.1038/srep44454 28327647 PMC5361117

[B81] Wells C. E. Eissenstat D. M. (2002). Beyond the roots of young seedlings: the influence of age and order on fine root physiology. *J. Plant Growth Regul.* 21 324–334. 10.1007/s00344-003-0011-1

[B82] Williams L. Carles C. Osmont K. S. Fletcher J. C. (2005). A database analysis method identifies an endogenous trans-acting short-interfering RNA that targets the *Arabidopsis* ARF2, ARF3, and ARF4 genes. *Proc. Natl. Acad. Sci. U.S.A.* 102 9703–9708. 10.1073/pnas.0504029102 15980147 PMC1172271

[B83] Willmann M. R. Poethig R. S. (2007). Conservation and evolution of miRNA regulatory programs in plant development. *Curr. Opin. Plant Biol.* 10 503–511. 10.1016/j.pbi.2007.07.004 17709279 PMC2080797

[B84] Wu G. Park M. Y. Conway S. R. Wang J. W. Weigel D. Poethig R. S. (2009). The sequential action of miR156 and miR172 regulates developmental timing in *Arabidopsis*. *Cell* 138 750–759. 10.1016/j.cell.2009.06.031 19703400 PMC2732587

[B85] Xie Q. Frugis G. Colgan D. Chua N. H. (2000). Arabidopsis NAC1 transduces auxin signal downstream of TIR1 to promote lateral root development. *Gene. Dev.* 14 3024–3036. 10.1101/gad.852200 11114891 PMC317103

[B86] Xing G. X. Zhu Z. L. (2000). An assessment of N loss from agricultural fields to the environment in China. *Nutr. Cycl. Agroecosys.* 57 67–73. 10.1023/A:1009717603427

[B87] Yan X. Liu X. Cui H. Zhao M. (2022). The roles of microRNAs in regulating root formation and growth in plants. *J. Integr. Agr.* 21 901–916. 10.1023/A:1009717603427

[B88] Yanagisawa S. Akiyama A. Kisaka H. Uchimiya H. Miwa T. (2004). Metabolic engineering with Dof1 transcription factor in plants: improved nitrogen assimilation and growth under low-nitrogen conditions. *Proc. Natl. Acad. Sci. U.S.A.* 101 7833–7838. 10.1073/pnas.0402267101 15136740 PMC419692

[B89] Yang J. H. Han S. J. Yoon E. K. Lee W. S. (2006). Evidence of an auxin signal pathway, microRNA167-ARF8-GH3, and its response to exogenous auxin in cultured rice cells. *Nucleic Acids Res.* 34 1892–1899. 10.1093/nar/gkl118 16598073 PMC1447648

[B90] York L. M. Lynch J. P. (2015). Intensive field phenotyping of maize (*Zea mays* L.) root crowns identifies phenes and phene integration associated with plant growth and nitrogen acquisition. *J. Exp. Bot.* 66 5493–5505. 10.1093/jxb/erv241 26041317 PMC4585417

[B91] Yu N. Niu Q. W. Ng K. H. Chua N. H. (2015). The role of miR156/SPLs modules in *Arabidopsis* lateral root development. *Plant J.* 83 673–685. 10.1111/tpj.12919 26096676

[B92] Zeng H. Wang G. Hu X. Wang H. Du L. Zhu Y. (2014). Role of microRNAs in plant responses to nutrient stress. *Plant Soil* 374 1005–1021. 10.1007/s11104-013-1907-6

[B93] Zhan A. Lynch J. P. (2015). Reduced frequency of lateral root branching improves N capture from low-N soils in maize. *J. Exp. Bot.* 66 2055–2065. 10.1093/jxb/erv007 25680794 PMC4378636

[B94] Zhang B. H. (2015). MicroRNA: a new target for improving plant tolerance to abiotic stress. *J. Exp. Bot.* 66 1749–1961. 10.1093/jxb/erv013 25697792 PMC4669559

[B95] Zhang B. H. Pan X. P. Cobb G. P. Anderson T. A. (2006a). Plant microRNA: a small regulatory molecule with big impact. *Dev. Biol.* 289 3–16. 10.1016/j.ydbio.2005.10.036 16325172

[B96] Zhang B. H. Pan X. P. Cobb G. P. Anderson T. A. (2006b). Conservation and divergence of plant microRNA gene. *Plant J.* 46 243–259. 10.1111/j.1365-313X.2006.02697.x 16623887

[B97] Zhang Q. (2007). Strategies for developing green super rice. *Proc. Natl. Acad. Sci. U.S.A.* 104 16402–16409. 10.1073/pnas.0708013104 17923667 PMC2034246

[B98] Zhang X. H. Zou Z. Zhang J. H. Zhang Y. Y. Han Q. Q. Hu T. X. (2011). Over-expression of sly-miR156a in tomato results in multiple vegetative and reproductive trait alterations and partial phenocopy of the sft mutant. *FEBS Lett.* 585 435–439. 10.1016/j.febslet.2010.12.036 21187095

[B99] Zhao M. Ding H. Zhu J. K. Zhang F. Li W. X. (2011). Involvement of miR169 in the nitrogen-starvation responses in *Arabidopsis*. *New Phytol.* 190 906–915. 10.1111/j.1469-8137.2011.03647.x 21348874 PMC3586203

[B100] Zhou Y. Liu X. Engstrom E. M. Nimchuk Z. L. Pruneda-Paz J. L. Tarr P. T. (2015). Control of plant stem cell function by conserved interacting transcriptional regulators. *Nature* 517 377–380. 10.1038/nature13853 25363783 PMC4297503

